# Wound of the Skull

**Published:** 1853-01

**Authors:** 


### ARTICLE XIX.

### *Wound of the Skull.*

The two following cases came under the care of M. Blandin, at the Hotel Dieu:

A bullet passed obliquely from right to left over the parietal bones, which were fractured in the extent of two inches and a half, the dura mater being injured. A considerable depression remained in the corresponding part of the head; complete paralysis of the lower, and incomplete loss of power of the superior, extremities followed. The patient was a man, aged thirty, of weak constitution. After unsuccessful endeavors to raise the depressed fragments, M. Blandin, applied the trephine on the right side of the mesial line. The fragments of bone were raised, and some of them removed, the dura mater was lacerated, and the surface of the brain was found in a contused state. Soon after the operation, the paralysis diminished in the left leg, and disappeared from the arms. Venesection was repeatedly employed. The wounds, at present in full suppuration, are dressed with cerate, lint, and over all, with a bladder half filled with ice; a portion of brain, of the size of a walnut, protrudes through the orifice. On the sixth day after the operation, the patient accidently fell out of bed, and abundant hemorrhage occurred. We will return to this case, the issue of which, must still remain doubtful.

The chin and branches of the inferior maxillary, were, in another patient, completely shot away, leaving an enormous irregular gap, through which the tongue hung downwards in a painful and swollen state. The dressing employed in this frightful mutilation, consisted of bands of adhesive plaster, which supported the lacerated edges of the cavity, and the tongue. This organ remains exquisitely painful, the suppuration is abundant, and hopes are entertained of the preservation of life.

